# Neurochemical effects of sleep deprivation in the hippocampus of the pilocarpine-induced rat model of epilepsy

**DOI:** 10.22038/ijbms.2020.50621.

**Published:** 2021-01

**Authors:** Heba S. Aboul Ezz, Aboul Ezz Noor, Iman M. Mourad, Heba Fahmy, Yasser A. Khadrawy

**Affiliations:** 1 Zoology Department, Faculty of Science, Cairo University, Giza, Egypt; 2 Biophysics Department, Faculty of Science, Cairo University, Giza, Egypt; 3 Medical Physiology Department, Medical Division, National Research Center, Giza, Egypt

**Keywords:** Amino acids, Cytokines, Epilepsy, Hippocampus, Oxidative stress, Sleep deprivation

## Abstract

**Objective(s):**

The present study aims to investigate the pathological mechanisms mediating the effect of paradoxical sleep deprivation (PSD) for 48 hr on the spontaneous recurrent seizures (SRS) stage of the pilocarpine rat model of temporal lobe epilepsy.

**Materials and Methods:**

This was carried out through assessment of amino acid neurotransmitter levels, the main oxidative stress parameters, and the levels of tumor necrosis factor-α (TNF-α), interleukin-1β (IL-1β), and interleukin-6 (IL-6) in the hippocampus. The experimental animals were divided into 4 groups: control, epileptic, PSD, and epileptic+PSD groups.

**Results:**

Data indicated that PSD in epileptic rats induced a significant decrease in GSH levels. TNF-α increased significantly in the PSD group and decreased significantly in both epileptic rats and epileptic rats deprived of paradoxical sleep. PSD induced a significant increase in glutamine, glutamate, and aspartate and a significant decrease in GABA. In epileptic rats and epileptic rats deprived of PS, a significant increase in aspartate and a significant decrease in GABA and taurine were recorded.

**Conclusion:**

The present data suggest that exposure to PSD for 48 hr did not worsen the alterations produced in the present epileptic model. However, epileptic, PSD, epileptic + PSD groups showed a state of hyperexcitability and oxidative stress. PSD may increase the susceptibility of animals to the development of epilepsy.

## Introduction

Epilepsy is a serious neurological disorder characterized by recurrent, unprovoked seizures (1). The pathogenesis of epilepsy and neuronal hyperexcitability has been linked with an imbalance between inhibitory and excitatory neurotransmission (2)^.^ In addition, oxidative stress has also been implicated in epileptogenesis (3). Epileptic seizures were also shown to induce an inflammatory response in the brain, by activation of microglia and release of proinflammatory cytokines. It has been demonstrated that brain pro-inflammatory cytokines are involved in the development and maintenance of seizures and the establishment of chronic epileptic foci (4).

Epilepsy exacerbates the risk of numerous comorbid conditions including sleep disorders (5). On the other hand, sleep deprivation is considered one of the principle triggering causes of seizures in epileptic patients (6). Insufficient sleep can further decrease the quality of life in epileptic patients due to the other comorbid conditions, especially cognitive impairments and/or psychological disorders, which also depend on sleep (7,8).

Sleep deprivation has been reported to induce oxidative stress leading to the generation of free radicals and neuronal damage (9). In addition, rapid eye movement sleep (REM) deprivation influences neurotransmitter levels, as well as other physiological and psychological processes (10). The immune system affects sleep patterns, under both physiological and pathological conditions (11) probably by the release of cytokines from immune cells in the brain or periphery (12).

The literature highlights a complex interaction between epilepsy and sleep. In spite of the great advance in our understanding of the mechanisms underlying the physiology of sleep and pathophysiology of epilepsy, there are still numerous unexplored or poorly understood areas (13). The most widely accepted proposal suggests that there is a reciprocal relationship between sleep and epilepsy and indicates an excitatory effect for sleep deprivation on some types of epilepsy (14).

The hippocampus is a limbic structure involved in learning and memory and the site of seizure initiation in the chronic phase of the rodent pilocarpine model (15, 16). Paradoxical sleep deprivation (PSD) affects learning and memory in both humans and animals (17). Thus, the present study aims to investigate the pathological mechanisms occurring in the hippocampus under the effect of PSD in the pilocarpine rat model of epilepsy. This was carried out through the assessment of amino acid neurotransmitter levels, the main oxidative stress parameters, and the levels of three inflammatory cytokines, namely, tumor necrosis factor-α (TNF-α), interleukin-1β (IL-1β), and interleukin-6 (IL-6) in pilocarpine-induced epileptic rats subjected to PSD for 48 hr during the spontaneous recurrent seizures (SRS) stage.

## Materials and Methods

***Experimental animals***

The experimental animals used in the present study were adult male Wistar albino rats weighing 200–250 g. They were given a standard laboratory diet and water *ad libitum*. The animals were kept under fixed conditions of housing and handling. Experimental protocols and procedures used in this study were approved by the Institutional Animal Care and Use Committee of Cairo University, Faculty of Science (IACUC) (Egypt), (CUFS/F/06/13). 

***Chemicals***

Pilocarpine was obtained from Macfarlan Smith Ltd. (Edinburgh, United Kingdom); it was dissolved in normal saline solution (0.9%). Atropine sulfate was obtained from Boehringer Ingelheim (Germany). Analytical-grade lithium carbonate, dansyl chloride, glacial acetic acid, and high-performance liquid chromatography (HPLC)-grade acetonitrile were obtained from Fisher (UK). Free amino acids for standard and HPLC-grade methanol were purchased from BDH (England). All other reagents were analytical grade reagents purchased from Sigma Chemical Co. (St. Louis, MO, USA).

***Induction of temporal lobe epilepsy***

Epilepsy was induced in rats by a single intraperitoneal (IP) injection of pilocarpine (380 mg/kg) as previously described (18). The animals were injected subcutaneously with atropine sulfate (5 mg/kg) 30 min before pilocarpine injection to prevent peripheral muscarinic stimulation (19). Then, the animals were left for 22 days to establish the chronic spontaneous recurrent seizures (SRS) phase of epilepsy (20).

***Induction of paradoxical sleep deprivation (PSD)***

PSD was induced using the multiple platform method to reduce immobilization stress (21). The animals were placed in plastic cages (100 cm × 50 cm × 50 cm) equipped with platforms and filled with water to 1 cm below the platform. Two platforms of different sizes were used; one with a large diameter (15 cm) for control animals in one cage and the other with a small diameter (3 cm) suitable for sleep-deprived animals in the other cage. When the animal enters the paradoxical phase of sleep, it falls into the water due to muscle atonia and wakes up. Food and water were available through a grid on the top of the cage containing water.

***Experimental design***

The experimental animals were divided into 4 groups (8 animals in each group). Animals of the 1^st^ group (epileptic group) were subjected to chronic epilepsy by receiving a single IP injection of pilocarpine. On the 22^nd ^day after epilepsy induction, they were placed on the platforms with large diameters for 48 hr to allow them to sleep normally. Animals of the 2nd group (PSD group) received an IP saline injection and on the 22^nd ^ day, they were placed for 48 hr on the small platforms to be subjected to PSD. Animals of the 3^rd^ group (epileptic + PSD group) were subjected to chronic epilepsy induction and on the 22^nd^ day, they were placed for 48 hr on the small diameter platforms for the induction of PSD. The 4^th^ group represented the control animals (control group) which were intraperitoneally injected with saline and after 22 days spent 48 hr on the large diameter platforms to sleep normally. All animals were sacrificed after 48 hr; the brain of each animal was dissected to remove the hippocampus. Each brain sample was divided into 2 halves; one half was used for the determination of amino acid neurotransmitters and the other half for the determination of oxidative stress parameters, AChE activity, and cytokine concentrations. The brain samples were weighed and kept at −20 °C until analyzed. 

***Determination of amino acid neurotransmitters***

The right half of the hippocampus of each animal was homogenized in 75 % ethanol and centrifuged at 15,777 × g at 4 °C in a high-speed cooling centrifuge (Type 3k-30, Sigma, Germany) for 30 min. The clear supernatant was evaporated to dryness and used for dansyl derivatization. HPLC was used for the quantitative determination of the amino acids (glutamate, aspartate, glutamine, GABA, glycine, and taurine) (22). The HPLC system consisted of a Wellchrom Mini-star K-501 pump (Knauer, Germany), a column thermostat equipped with a 20 μl loop injector (Knauer, Germany), a luna 5u C-18 reversed-phase column (5 μm particle size, 150 × 4.6 mm I.D.) from Phenomenex, USA, a Wellchrom spectrophotometer K-2600 (Knauer, Germany) and a chromatography workstation (Eurochrom 2000). The mobile phase consisted of 50/50 (v/v), methanol/water containing 0.6% glacial acetic acid, and 0.008% triethylamine. The flow rate was 1 ml/min and the wavelength was 254 nm. Amino acid concentrations were determined by the internal standard method using 2-aminobutyric acid as an internal standard and were expressed as μmol/g fresh tissue.

***Determination of oxidative stress parameters, cytokine levels, and AChE activity***

Each brain sample was homogenized in 5% w/v 20 mM phosphate buffer, pH 7.6. Then the homogenates were centrifuged at 15777 g, 4 °C in a high-speed cooling centrifuge for 15 min. The clear supernatant was used for the assay.

***Determination of lipid peroxidation***

Malondialdehyde (MDA) was used as an index to measure lipid peroxidation (23). The thiobarbituric acid reactive substances react with thiobarbituric acid to produce a pink colored complex whose absorbance was read at 532 nm in a Helios Alpha Thermospectronic (UVA 111615, England).

***Determination of Nitric Oxide (NO) level***

Nitric oxide (NO) level was measured as nitrite (24). Using Griess reagent, nitrite is converted to a deep purple azo compound, the absorbance of which was measured spectrophotometrically at 450 nm.

***Determination of reduced glutathione (GSH) level***

The determination of reduced glutathione (GSH) was based on Ellman’s method (25). The -SH groups of GSH reduce Ellman’s reagent forming 2-nitro-s-mercaptobenzoic acid. This anion has an intense yellow color whose absorbance was measured spectrophotometrically at 412 nm.

***Determination of AChE activity***

The method used for determination of AChE activity in the hippocampus was a modification of the procedure of Ellman *et al.* (26, 27). The method is based on measuring the thiocholine produced after the hydrolysis of acetylthiocholine. Thiocholine reacts with the -SH reagent 5,5’-dithiobis-(2-nitrobenzoicacid) (DTNB), reducing it to thionitrobenzoic acid, which is a yellow colored anion whose absorption was read immediately at 412 nm.

***Determination of TNF-α, IL-6, and IL-1β***

Estimation of TNF-α level in the hippocampus was carried out using rat TNF-α ELISA Kit supplied by Koma Biotech INC, Seoul (Korea). The quantification of IL-6 in the hippocampus was performed using Rat IL-6 ELISA Kit no.95053 (Glory Science Co., Ltd, USA). For the quantitative determination of IL-1β in the hippocampus, Rat IL-1β ELISA Kit no.30419 (Glory Science Co., Ltd, USA) was used. The concentrations of the three cytokines were then determined using standard curves. 

***Statistical analysis***

Data were analyzed by analysis of variance (ANOVA) followed by the Duncan multiple range test when the F-test was significant (*P*<0.05). All analyses were performed using the Statistical Package for Social Sciences (SPSS) software package on a compatible computer.

## Results

***Results of neurotransmitters***

As shown in Table 1, ANOVA revealed significant increases in hippocampal aspartate levels in epileptic, PSD, and epileptic + PSD groups in comparison with the control group. Glutamine and glutamate concentrations showed a significant increase in the PSD group only. On the other hand, GABA decreased significantly in epileptic, PSD, and epileptic + PSD groups with reference to the control group. Taurine levels revealed significant decreases in both epileptic animals and epileptic + PSD animals relative to control animals. No significant changes in hippocampal glycine levels were observed in the three studied groups.

***Results of oxidative stress***

**Figure 1:**
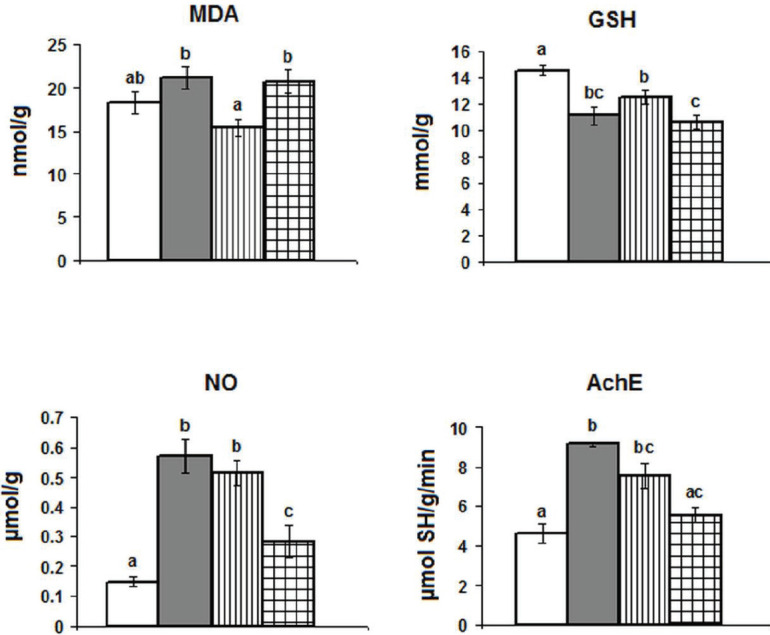
Effect of paradoxical sleep deprivation for 48 hr on the levels of lipid peroxidation (MDA), reduced glutathione (GSH), nitric oxide (NO), and acetylcholinesterase activity (AchE) in the hippocampus of control and epileptic rats

Regarding oxidative stress parameters (Figure 1), significant decreases were observed in GSH levels in the three groups in comparison with control rats. On the contrary, recorded NO levels showed significant increases in the three investigated groups. In addition, increased levels of MDA were recorded in both PSD and epileptic + PSD groups when compared with epileptic animals only. Acetylcholinesterase activity revealed significant increases in the rat model of epilepsy and in PSD rats relative to the control group.

***Results of inflammatory factors***

**Figure 2:**
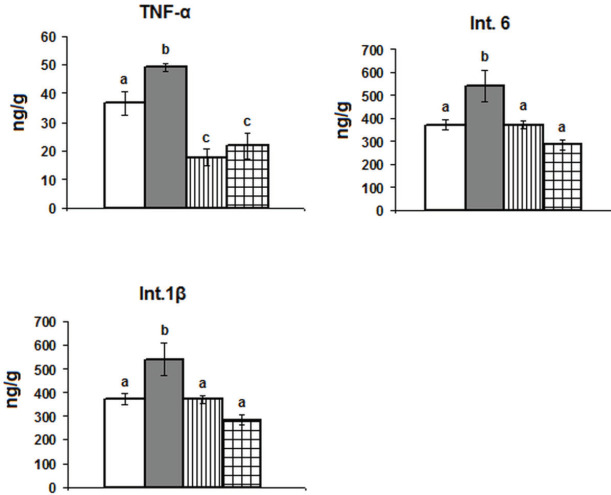
Effect of paradoxical sleep deprivation for 48 hr on the levels of tumor necrosis factor-α (TNF-α), interleukin-6 (Int.6), and interleukin 1β (Int.1β) in the hippocampus of control and epileptic rats

In comparison with control animals, TNF-α increased significantly in the PSD group and decreased significantly in both epileptic rats and epileptic rats deprived of paradoxical sleep. This was accompanied by significant increases in IL- 6 and IL-1β in the PSD group only (Figure 2).

Control

Paradoxical sleep (PS) deprived rats

Rat Model of epilepsy

Rat Model of epilepsy deprived of PS

Different letters indicate significantly different means at -value<0.05

**Table 1:** Effect of paradoxical sleep deprivation for 48 hr on the levels (µmol/g) of glutamine, glutamate, aspartate, γ-aminobutyric acid, glycine, and taurine in the hippocampus of control and epileptic rats

	Control	Sleep-deprived rats	%D	Epileptic rats	%D	Epileptic rats deprived of sleep	%D	*P-*value
								
Glutamine	3.354^a^ ± 0.249 (6)	4.112^b^ ± 0.266 (6)	+22.59	3.019^a^ ± 0.08 (8)	-9.98	3.543^a^ ± 0.06 (8)	+5.65	0.002
Glutamate	8.748^a^ ± 0.680 (6)	10.127^b^ ± 0.521 (6)	+15.76	7.935^a^ ± 0.265 (8)	-9.29	8.766^a^ ± 0.274 (7)	+0.21	0.009
Aspartate	6.156^a^ ± 0.460 (6)	7.780^b^ ± 0.154 (7)	+26.38	8.167^bc^ ± 0.109 (8)	+32.67	8.645^c^ ± 0.160 (6)	+40.43	0.000
GABA	3.210^a^ ± 0.174 (7)	2.832^b^ ± 0.042 (7)	-11.78	2.792^b^ ± 0.041 (7)	-13.02	2.818^b^± 0.162 (8)	-12.31	0.017
Glycine	1.238^a^ ± 0.053 (7)	1.199^a^ ± 0.066 (7)	-3.15	1.145^a^ ± 0.028 (6)	-7.51	1.106^a^ ± 0.027 (8)	-10.66	0.216
Taurine	4.992^a^ ± 0.245 (6)	5.051^a^ ± 0.111 (7)	+1.18	3.701^b^ ± 0.06 (8)	-25.86	3.818^b^ ± 0.162 (8)	-23.52	0.000

Values represent mean±SE with the number of animals between parentheses

% D: % difference with respect to control values.

Different letters indicate significantly different means at *P*-value<0.05

Same letters indicate nonsignificant changes

Control

Paradoxical sleep (PS) deprived rats

Rat Model of epilepsy

Rat Model of epilepsy deprived of PS

Different letters indicate significantly different means at *P*-value<0.05

## Discussion

The present findings indicate a state of hyperexcitability in the hippocampus of 48 hr PSD rats mediated by the increased excitatory amino acids (glutamate and aspartate) and the reduced inhibitory amino acid (GABA). Similar results were obtained after 72 hr of PSD (28). This condition was associated with oxidative stress which was evident from the increased NO and the decreased GSH levels. Also, a state of neuroinflammation mediated by the increased inflammatory cytokines was observed. The hippocampus has a high density of N-methyl-D-aspartate receptors (29). When these receptors are activated by glutamate and aspartate, a massive influx of calcium ions occurs leading to the production of reactive oxygen and nitrogen species, a condition known as glutamate excitotoxicity which contributes to neuronal death (30). Besides, the increased expression of glutamine synthetase after PSD may favor the conversion of glutamate to glutamine to reduce the excitation (31), which explains the present increase in glutamine after PSD. However, this mechanism failed in controlling the state of hyperexcitability leading to oxidative stress. The reduced GSH may arise from its exhaustion in scavenging the free radicals. The present results agree with a previous study that reported an increase in oxidative stress in rats subjected to PSD (32).

Furthermore, the present increase in AChE activity indicates a decrease in cholinergic activity due to the enhanced degradation of ACh, an effect that is expected in view of the reported adverse effects of SD on memory and learning (17, 33).

Moreover, TNF-α can potentiate glutamate-mediated cytotoxicity indirectly by inhibiting glutamate transport on astrocytes, and directly by increasing the localization of ionotropic glutamate receptors to synapses (34). Thus, the present increase in TNF-α could exaggerate the state of hyperexcitability and consequently the oxidative stress. It has been reported that long-term SD (5 days) induced increased cerebral microglial activation (35). This produces O^˙¯2^, H_2_O_2_, and cytokines such as IL-1, IL-6, and TNF-α, the latter stimulating inducible NO synthase (iNOS) and forming excess NO. 

The epilepsy model induced by pilocarpine in rats is characterized by (1) an acute phase, characterized by seizures which progress within 1–2 hr to status epilepticus (SE), (2) a seizure-free silent period (4–44 days, mean of 15 days) during which no seizures occur, however, structural and neurochemical changes take place and (3) a chronic phase, characterized by spontaneous recurrent seizures (SRS) during which seizures occur at unexpected time intervals [10, 11]. Excitatory terminals in the hippocampus contain both glutamate and aspartate (36, 37). Thus, the increased aspartate and decreased GABA levels may result in a state of hyperexcitability that may in turn lead to the initiation of paroxysmal activity in the hippocampus. The nonsignificant changes in hippocampal glutamate during the SRS phase of epilepsy have been reported by another study which also found a significant increase in aspartate and a significant decrease in GABA in the hippocampus during the same phase (38). Evidence from epileptic animal and humans suggested that the dysfunction of inhibitory GABAergic circuits may contribute to the excitation/inhibition imbalance underlying the pathologic mechanism of epilepsy (39). In addition, taurine is known to prevent neuronal death during excitotoxicity by modulating the ability of the mitochondria to buffer intracellular calcium (40). Therefore, the present significant decrease in taurine may exaggerate the hippocampal excitotoxicity induced by pilocarpine thus precipitating the epileptic condition. 

Acetylcholine (ACh) plays a critical role in seizure activity (41). In the CNS, the excessive production or release of ACh may underlie the neuronal damage induced by pilocarpine during SE and SRS (42). Therefore, the present significant increase in AChE activity in the hippocampus of pilocarpine-treated rats during SRS may represent an attempt to terminate the increase in cholinergic hyperactivity as suggested previously (43).

Oxidative stress has been established as a possible mechanism in epileptogenesis and was found to underlie the neurochemical changes observed during SRS induced by pilocarpine (44). The present data of epileptic rats also showed a state of oxidative stress as indicated by the significant decrease in hippocampal GSH and the significant increase in NO. This could be attributed to the hyperexcitability induced by the imbalance between excitatory and inhibitory amino acids.

These results are in agreement with a previous study that reported a significant increase in NO levels and AChE activity accompanied by a significant decrease in GSH levels in the hippocampus of pilocarpine-treated rats during SRS (43). 

As it has been suggested that the increase in NO formation might provide a general mechanism for seizure initiation (45), the present elevation in NO levels may contribute to the development of seizures during SRS induced by pilocarpine. Interestingly, NO was found to reduce synaptosomal GABA uptake thereby decreasing the availability of GABA (46). This could explain the present decrease in GABA concentration in the pilocarpine model during SRS.

It was reported that low concentrations of TNF-α may activate proconvulsive effects via p55 while high concentrations of TNFα can play an anticonvulsive role through the p75 pathway (47). Therefore, the present decrease in TNF-α may mediate the sporadic seizures in the pilocarpine model of chronic epilepsy. Molecules such as IL-1β, TNF-α, and IL-6 are increased rapidly after seizure induction, decreasing to basal levels within 48–72 hr after seizure onset (48). Therefore, the present non-significant changes in IL-6 and IL-1β may be due to an adaptive mechanism in the chronic state of pilocarpine-induced epilepsy.

Sleep has been suggested to have an antioxidant function and to influence brain excitability especially in epilepsy (49). In the present investigation, PSD for 48 hr during the SRS phase of the rat model of epilepsy precipitated the hyperexcitability state mediated by increased aspartate and decreased GABA and taurine levels.

During REM sleep, the increasing GABAergic activity is protective of seizures (49). Therefore, the present decrease in GABA could be attributed to PSD which may exaggerate the epileptic activity in pilocarpine-treated rats. Also, the increased hippocampal excitability could result in lowering the seizure threshold making the animal susceptible to any stimuli and result in oxidative stress.

When the present pilocarpine-treated rats were deprived of PS for 48 hr, the significant decrease in GSH levels and significant increase in NO continued with no change in the MDA level. The present persistent increase in NO levels in PSD-epileptic animals could contribute to the reduced seizure threshold through its stimulatory effect on the excitatory amino acid neurotransmitters (50). The lowered GSH level may indicate its exhaustion in overcoming the evolved free radicals during PSD, SRS and SRS deprived of PSD. This could explain the nonsignificant decrease in lipid peroxidation in the epileptic rat model and its slight increase in PSD and epileptic rats deprived of PSD. Our results are in agreement with several studies that did not find any increase in lipid peroxidation with short-term sleep deprivation (9, 51).

It may be suggested that exposure of epileptic rats to PSD for 48 hr precipitated the state of oxidative stress without exacerbating it. However, it is clear that PSD for 48 hr increased MDA levels significantly in comparison with epileptic animals. 

Peculiarly, the recorded significant increase in AChE activity in each of the epileptic and PSD groups was abolished in epileptic animals subjected to PSD. Evidence suggests that inhibition of AChE is mediated by oxidative stress (52). It has been reported that AChE is very sensitive to free radicals and that hydroxyl radicals are involved in AChE inhibition (53). The decrease in AChE activity is probably related to the increase in MDA levels in PSD-epileptic rats. This inhibition may lead to the facilitation of seizure activity if the duration of PSD was prolonged since it would ultimately lead to an increase in cholinergic activity.

Unexpectedly, induction of PSD for 48 hr in the SRS phase of the present pilocarpinized rats resulted in non-significant changes in the three studied cytokines. This may be attributed to the reciprocal effects of epilepsy and PSD on these cytokines as clear from the decrease obtained in TNF-α during the chronic phase of the pilocarpine model and the increase in TNF-α, IL-6, and IL-1β in PSD animals.

## Conclusion

The present data indicate that PSD in the rat model of temporal lobe epilepsy induced hippocampal oxidative stress and increased the hippocampal excitability by shifting the equilibrium between the excitatory and inhibitory amino acid neurotransmitters towards excitation. This effect could contribute to the reduction of seizure threshold in the present rat model of epilepsy and make the animals prone to convulsions.

The present study may provide a starting point for future research on this adverse association between epilepsy and PSD and raise awareness of the importance of sufficient sleep in epileptic patients.
